# KPT-330 inhibition of chromosome region maintenance 1 is cytotoxic and sensitizes chronic myeloid leukemia to Imatinib

**DOI:** 10.1038/s41420-018-0049-2

**Published:** 2018-04-23

**Authors:** Danian Nie, Kezhi Huang, Songmei Yin, Yiqing Li, Shuangfeng Xie, Liping Ma, Xiuju Wang, Yudan Wu, Jie Xiao, Jieyu Wang, Wenjuan Yang, Hongyun Liu

**Affiliations:** 1 Guangdong Provincial Key Laboratory of Malignant Tumor Epigenetics and Gene Regulation and Department of Hematology, Sun Yat-Sen Memorial Hospital, Sun Yat-Sen University, 510120 Guangzhou, China; 2 Department of Hematology, Hemostasis, Oncology, and Stem Cell Transplantation, Hannover Medical School, Carl-Neuberg-Straße 1, 30625 Hannover, Germany; 3 Department of Translational Molecular Pathology, The University of Texas MD Anderson Cancer Center, 2130 West Holcombe Boulevard, Suite 910, Houston, TX 77030 USA

## Abstract

As tyrosine kinase inhibitors (e.g., Imatinib, IM) fail to induce long-term response in some chronic myeloid leukemia (CML), novel therapies targeting leukemia-dysregulated pathways are necessary. Nuclear-cytoplasmic trafficking of proteins play a key role in the development of leukemia and drug resistance. KPT-330 (Selinexor), an inhibitor of chromosome region maintenance 1 (CRM1, nuclear receptor exportin 1, XPO1), demonstrated activities against a few hematological malignancies. We examined the anti-leukemic efficacy of KPT-330 in IM-resistant CML. Cell viability was examined by MTS assay. Apoptosis and cell cycle were assessed by flow cytometry. CRM1 mRNA was detected by PCR. Expression of CRM1 protein and its cargo proteins were determined by western blot or immunofluorescent staining. Furthermore, we engrafted nude mice subcutaneously with IM-resistant CML K562G. Mice were treated with IM, KPT-330 alone or in combination. Expression of CRM1 in CML were markedly higher than control. KPT-330 inhibited proliferation, induced cell cycle arrest and apoptosis of K562 and K562G. IC50 of IM on K562G was reduced by KPT-330. Mechanistically, KPT-330 inhibited CRM1 and increased the nuclear/cytoplasm ratio of BCR-ABL and P27. p-AKT was downregulated while p-STAT1 and caspase-3 were upregulated. Furthermore, KPT-330 showed anti-leukemic effect in primary IM-resistant CML with T315I mutation in CRM1-dependent manner. In K562G xenograft mice model, KPT-330 inhibited tumor growth and sensitized K562G to IM in vivo. To conclude, KPT-330 showed anti-leukemic activity and sensitized CML to IM in CRM1-dependent manner in vitro and in vivo. KPT-330 represents an alternative therapy for IM-refractory CML, warranting further investigation of CRM1 as therapeutic target.

## Introduction

Chronic myeloid leukemia (CML), a clonal myeloproliferative disorder, is characterized by the Philadelphia chromosome (Ph), which is generated by the translocation of chromosomes 9 and 22^1^. This cytogenetic aberrance forms a constitutively active tyrosine kinase, the BCR-ABL chimeric oncogene. BCR-ABL is exported to the cell cytoplasm where it performs the proliferation and anti-apoptotic capacities via activation of dual signaling pathways, facilitating the expansion of leukemic myeloid cells^2^.

Targeting BCR-ABL has been validated as a powerful strategy for fighting against CML. Imatinib (IM), the specific inhibitor for BCR-ABL kinase, has opened a new era for treating CML. Nowadays, IM is considered as the first-line treatment for CML in chronic phase and even some more advanced stages. However, resistance to this drug develops in about 30% of patients via various mechanisms, including BCR-ABL gene mutations (e.g., T315I), gene amplifications and activation of other signaling pathways such as PI3K-AKT, JAK-STAT^3,4^. Although newer generation of BCR-ABL inhibitors have been developed, other therapeutic targets are also urgently needed and proposed^5,6^.

Chromosome maintenance protein 1 (CRM1; XPO1, Exportin-1), is an important nuclear protein export receptor transporting target proteins across a Ran-GTP gradient^7,8^. CRM1 is involved in the transport of a variety of cargo proteins, such as tumor suppressor proteins (e.g., STAT1), cell-cycle regulators (e.g., P27^9^,) and tyrosine kinases (e.g., BCR-ABL)^10^. Overexpression of CRM1 has been detected in different tumors and correlates with adverse outcome^11–14^. Therefore, modulation of CRM1-mediated nuclear export of signaling molecules presents as an attractive antineoplastic, including anti-leukemic therapeutic approach^12,15,16^, and even for addressing drug-resistance^10^. However, the older CRM1 inhibitors, such as leptomycin B (LMB), have severe 'off-target' cytotoxicity, limiting their clinical application^17^. The selective inhibitors of nuclear export (SINE) are currently developing and have shown clinical efficacy against malignancies with fewer side effect. KPT-330 (Selinexor), an oral bioavailable clinical stage SINE class of CRM1 antagonists, demonstrated activities against several solid and hematological cancers including acute myeloid leukemia (AML) and acute lymphoblastic leukemia (ALL)^18,19^. Importantly, KPT-330 eradicated the leukemia-initiating cells (LICs) of AML and reversed the inherent drug-resistant in murine model and phase I/II clinical trials^20^(NCT02249091, NCT02088541, NCT02093403, and NCT02299518). However, very few studies shed light on the effect of KPT-330 on CML.

In this work, we characterized the biologic activity of CRM1 inhibitor KPT-330 in CML cell lines, patient blasts, and in a murine xenograft CML model. KPT-330 showed remarkable anti-proliferative and pro-apoptotic properties against CML cell lines and patient blasts, including those from patients with BCR-ABL T315I mutation resistant to IM. Mechanistically, KPT-330 treatment restored the localization of cytoplasmic BCR-ABL and P27 into the nucleus, downregulated p-AKT and upregulated p-STAT1 and caspase-3 in a CRM1-dependent manner. Furthermore, we showed in vivo the anti-leukemia potency of KPT-330 in a murine CML model bearing the K562G resistant mutant.

## Results

### High expression of CRM1 in K562, K562G and primary CML cells

**Fig. 1:**
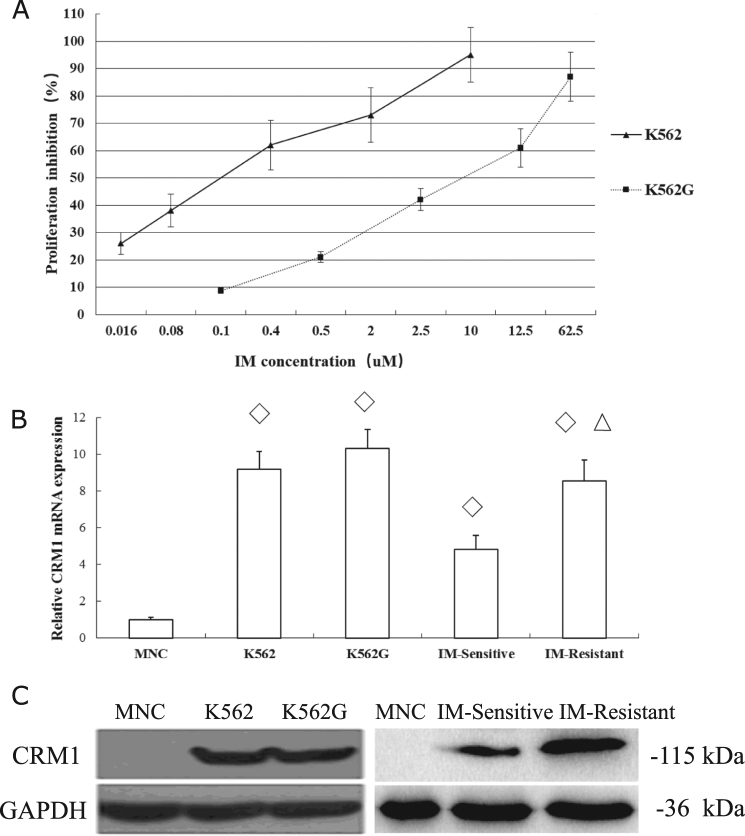
CRM1 was highly expressed in K562, K562G, and primary CML cells. **a** K562G was highly resistant to IM compared with K562 after incubation for 48 h in MTS assay. The mRNA and protein expression of CRM1 in CML cells were significantly higher than normal MNC, as indicated by real-time PCR (**b**) and western blot (**c**). ◇Compared with MNC, *P* < 0.05, Student’s *t*-test; △Compared with IM-sensitive primary CML cells, *P* < 0.05, Student’s *t*-test. Data were expressed as mean ± SD. Results were representative of at least 2 independent experiments. MNC, mononuclear cells

K562G cell was derived from K562, a human cell line of CML positive for BCR-ABL and widely used for leukemia research. K562G was established by using escalating concentrations of IM and gained resistance to IM over several months of culture as described^21^. To qualify the K562G cell line in our study, the half maximal inhibitory concentration (IC50) of K562G to IM was examined. K562G showed high resistance to IM with 27.87-fold compared to K562 (IC50: 4.46 vs 0.16 μM, *P* < 0.05. Figure 1a), similar to previous report^22^. The expression of CRM1 was determined in MNC of normal bone marrow, K562, K562G, IM-sensitive, and IM-resistant primary CML cells, respectively. In both mRNA (Fig. 1b) and protein level (Fig. 1c), the CRM1 expression of CML cell line or primary CML cells was significantly higher than that in normal MNC. While there was no difference between K562 and K562G for CRM1 expression, the IM-resistant primary CML cell showed markedly higher CRM1 expression than IM-sensitive primary cells (Fig. 1b, c). The data indicated that CRM1 was highly expressed in CML particularly those with IM-resistance and might be a potential therapeutic target.

### KPT-330 showed proliferation inhibition, cell cycle arrest, and apoptosis induction in K562 and K562G cells

**Fig. 2:**
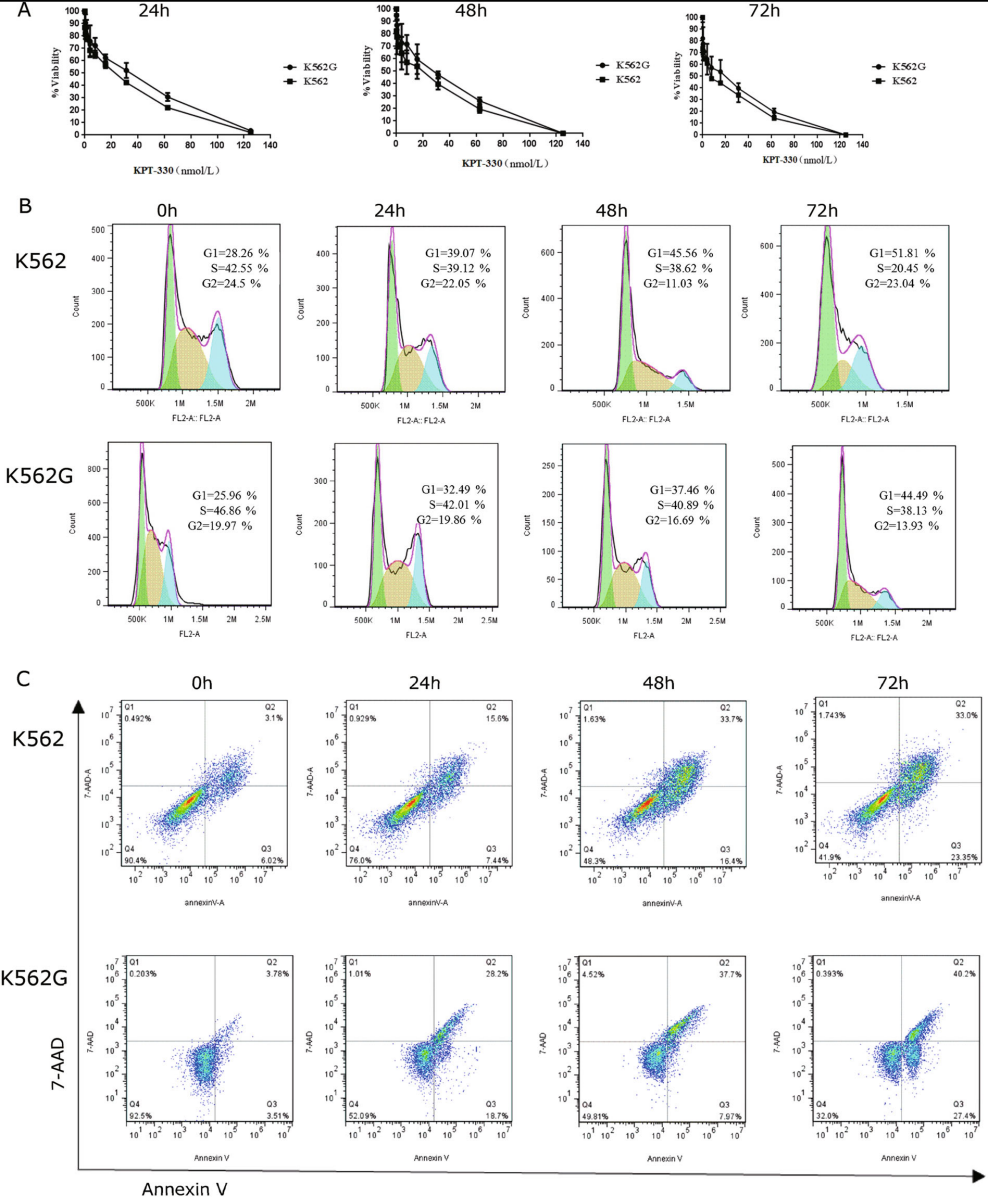
KPT-330 inhibited cell proliferation, arrested cell cycle, and induced apoptosis in K562 and K562G cells. **a** Cells were incubated with different concentrations of KPT-330 in 96-well plate for 24, 48 and 72 h. MTS assay was performed to determine the proliferation inhibition. IC50 of K562G were only slightly higher than that of K562, indicating KPT-330 was potent to inhibit cell proliferation in both K562 and K562G. **b** Cell cycle was analyzed by flow cytometry after KPT-330 exposure. The proportion of G1 phase was increased while S and G2 phase decreased in both K562 (0.1 nM KPT-330) and K562G (1.3 nM KPT-330). **c** KPT-330 triggered remarkable apoptosis in both K562 (12 nM KPT-330) and K562G (23 nM KPT-330) as shown by Annexin-V/7-AAD staining. Results were representative of 3 independent experiments

KPT-330, the CRM1-selective inhibitor of nuclear export (SINE), was used to test the anti-leukemic activity in K562 and K562G cells. Firstly, KPT-330 inhibited proliferation of K562 and K562G cells in a time and dose-dependent manner, as shown in the morphology analysis (data not shown) and MTS assay (Fig. 2a). At the time point of 24, 48, and 72 h, the IC50 of KPT-330 for K562G was 33.25 nM, 23.69 nM, and 15.56 nM, which were only slightly higher than that for K562 (18.79 nM,14.44 nM, and 7.78 nM), implying that KPT-330 was potent to inhibit cell proliferation of both K562 and K562G. Next, the cell cycle distribution was analyzed by flow cytometry after KPT-330 treatment. As shown in the Fig. 2b, the proportion of G1 phase for K562 cell was increased while S and G2 phase decreased with KPT-330 treatment. Similar results were obtained for K562G cells (Fig. 2b), suggesting that KPT-330 arrested both IM-sensitive and IM-resistant CML cells in G1 phase cell cycle. In addition, the apoptosis rate of K562 cell was significantly higher than control after 48 h of 12 nM KPT-330 exposure (50.15 ± 4.66% vs 9.12 ± 0.78%, *P* < 0.05, Fig. 2c). Similarly, 48 h of 23 nM KPT-330 exposure led to remarkable cell apoptosis for K562G compared with untreated group (45.67 ± 3.25% vs 7.29 ± 0.88%, *P* < 0.05, Fig. 2c). These data indicated that KPT-330 had prominent anti-leukemic activity in both K562 and K562G cells: proliferation inhibition, cell cycle arrest, and apoptosis induction.

### KPT-330 sensitized K562G to IM

The use of IM for CML remains the first-line treatment in the tyrosine kinase inhibitor (TKI) era. Sensitization of resistant CML cells to TKI is of high significance in clinic. In the cytotoxic assay, we had found there was almost no inhibition for K562G cell growth using 0.1 nM KPT-330. Hence, low concentrations of KPT-330 (0.05 nM, 0.1 nM, and 0.2 nM) were added to test their effect on sensitization of K562G to IM. After addition of low concentrations of KPT-330 as indicated above for 48 h, the IC50 of IM for K562G were significantly reduced (Table 1). The data suggested that low concentration of KPT-330 sensitized K562G to IM.

**Table 1:** KPT-330 sensitized K562G to IM

IC50 of IM with low concentrations of KPT-330 for K562G		
Group	IC50 (μM)	IC50_IM_/IC50_KPT-330_ _+_ _IM_
IM	5.099	
KPT-330(0.05 nM) + IM	3.893	1.310
KPT-330(0.1 nM) + IM	2.494	2.045
KPT-330(0.2 nM) + IM	2.296	2.221

Low concentrations of KPT-330 were added with IM to enhance proliferation inhibition of K562G. IC50 of IM decreased as the concentrations of KPT-330 increased from 0.05 to 0.2 nM, which were nearly not toxic toward K562G. IC50 were calculated from MTS cytotoxic assay by SPSS 17.0 software. *IC50* half maximal inhibitory concentration, *IM* Imatinib. Results were representative of 3 independent experiments

### KPT-330 increased the nuclear/cytoplasm ratio of BCR-ABL and P27, downregulated p-AKT and upregulated p-STAT1 and cleaved caspase-3 in CRM1-dependent manner

**Fig. 3:**
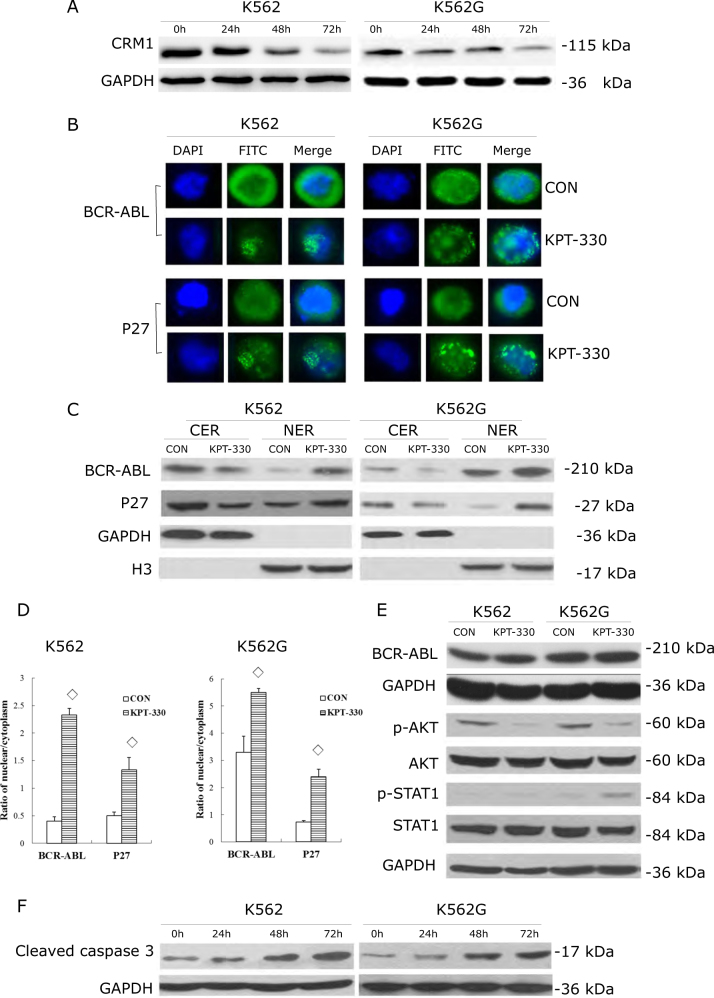
KPT-330 increased the nucleic/cytoplasm ratio of BCR-ABL and P27, downregulated p-AKT and upregulated p-STAT1 and cleaved caspase-3 in CRM1-dependent manner. K562 was treated with 0.1 nM KPT-330 and K562G with 1.3 nM KPT-330 for 48 h, respectively. **a** CRM1 was inhibited by KPT-330 in both K562 and K562G in time-dependent manner as shown by western blot. Immunofluorescence (**b**) and western blot (**c**) showed the nuclear/cytoplasm ratio of BCR-ABL and P27 increased after KPT-330 exposure. **b** BCR-ABL and P27 were highly increased in nuclear and decreased in cytoplasm by KPT-330 observed under fluorescent microscope (×100). This was further confirmed by western blot analysis using subcellular fractions of lysates (**c**). The ratio of nuclear/cytoplasm of BCR-ABL and P27 after KPT-330 exposure were significantly higher than control (**d**). However, KPT-330 did not affect the whole expression of BCR-ABL (**e**). p-AKT (**e**) was downregulated while p-STAT1 (**e**) and cleaved caspase-3 (**f**) were upregulated revealed by western blot. GAPDH was used as internal control for whole protein/CER and H3 for NER. CER cytoplasmic extraction reagent, NER nuclear extraction reagent. ◊ compared with control, *P* < 0.05, Student’s *t*-test. Data were expressed as mean ± SD. Data were representative of 3 independent experiments

To obtain insights about the mechanisms by which KPT-330 had anti-leukemic effect, CRM1 and its cargo-protein were further studied. Firstly, the western blot data clearly showed CRM1 was inhibited by KPT-330 in both K562 and K562G cells (Fig. 3a). As the cargo-protein of CRM1, BCR-ABL and P27 could be transported from nuclear to cytoplasm by CRM1. The aberrant location of these cargo proteins led to the abnormal cell proliferation, cell cycle distribution and apoptosis, which were the significant events in the development of CML. Immunofluorescence and western blot were used to determine the distribution and nuclear/cytoplasm ratio of BCR-ABL and P27 after KPT-330 exposure. As shown in Fig. 3b, BCR-ABL and P27 were highly increased in nuclear and decreased in cytoplasm by KPT-330 in both K562 and K562G cells under fluorescent microscope. This was further confirmed by western blot analysis using subcellular fractions of lysates (Fig. 3c). The ratio of nuclear/cytoplasm of BCR-ABL and P27 were significantly elevated (Fig. 3d). To see whether KPT-330 had effect on the overall expression of BCR-ABL, the whole protein was extracted and analyzed. Not surprisingly, KPT-330 did not affect the whole expression of BCR-ABL (Fig. 3e), underlining its unique action on CRM1 and the cargo proteins.

AKT and STAT1 play key role in cell proliferation and signaling transduction. Next, the phosphorylation of AKT and STAT1 were examined. After treatment with KPT-330 with indicating concentrations, p-AKT was downregulated while p-STAT1 was upregulated in both K562 and K562G cells (Fig. 3e). Since KPT-330 showed prominent induction of apoptosis in CML cells as shown above (Fig. 2c), caspase-3 dependent apoptosis pathway was studied. Cleaved caspase-3 expression was found to be much higher after KPT-330 treatment (Fig. 3f), suggesting that KPT-330 induced cell apoptosis at least partly via caspase-3 dependent pathway. Thus, KPT-330 inhibited CML cell growth, triggered cell cycle arrest and apoptosis by entrapping BCR-ABL and P27 in the nucleus, inhibiting the activities of oncogenes like AKT and activating tumor suppress protein like STAT1 and apoptosis inducer caspase-3 (Figure 3).

### KPT-330 showed anti-leukemic effect on primary IM-resistant CML in CRM1-dependent manner

**Fig. 4:**
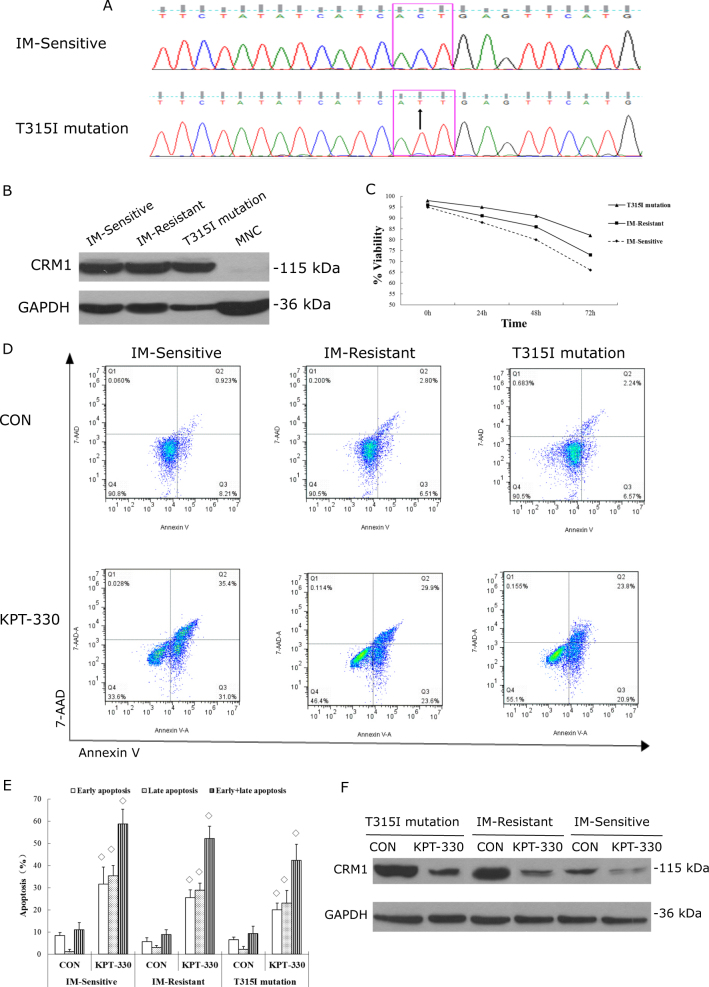
KPT-330 exhibited anti-leukemic effect on primary IM-resistant CML in CRM1-dependent manner. **a** Sequencing data showed primary CML cells without (IM- Sensitive) or with ABL T315I mutation. **b** Western blot showed CRM1 protein were highly expressed in the primary CML cells, especially in the T315I-mutated cells. IM-sensitive CML was treated with 0.1 nM KPT-330 and IM-Resistant (with or without T315I mutation) CML with 1.3 nM KPT-330, respectively. **c** KPT-330 inhibited primary CML growth in time-dependent manner. Apoptosis was induced not only in IM-sensitive, but also in IM-Resistant (with or without T315I mutation) CML cells after KPT-330 treatment for 48 h (**d**, **e**). **f** Exposure to KPT-330 for 48 h significantly reduced CRM1 expression in primary CML as shown by western blot. ◊ compared with control, *P* < 0.05, Student’s *t*-test. Data were expressed as mean ± SD. Shown were representative data of 3 independent experiments

To better understand the anti-leukemic effect of KPT-330 on CML, the primary CML cells which were IM-sensitive, IM-resistant without T315I mutation or IM-resistant with T315I mutation were used. The three patients were all diagnosed as CML by morphology, immunology, cytogenetics, and molecular analysis. During the treatment course with IM, one responded well and achieved complete remission (IM-sensitive), the other two did not show response to IM without or with T315I mutation (Fig. 4a). In detail, the one with T315I mutation presented with enormous leukocytosis (WBC 809 × 10^9^/L) with ratio of BCR-ABL/ABL 0.443 at diagnosis. The patient did not respond to IM, the second-generation TKI dasatinib and traditional chemotherapy, with a survival of 5 months.

CRM1 protein were highly expressed in the three primary CML cells, particularly in the T315I-mutated cells (Fig. 4b). Based on the experience of K562 and K562G experiments, we chose 0.1 nM and 1.3 nM of KPT-330 for proliferation/apoptosis assays, for IM-sensitive and IM-resistant primary CML cells, respectively. As shown in Fig. 4, KPT-330 were potent to inhibit cell growth (Fig. 4c) and induce apoptosis (Fig. 4d, e) not only in IM-sensitive, but also in IM-resistant (with or without T315I mutation) CML cells. Furthermore, CRM1 expression were decreased after KPT-330 exposure (Fig. 4f), suggesting the anti-leukemic effect of KPT-330 was CRM1-dependent.

### KPT-330 inhibited tumor growth and sensitized K562G to IM in mice

**Fig. 5:**
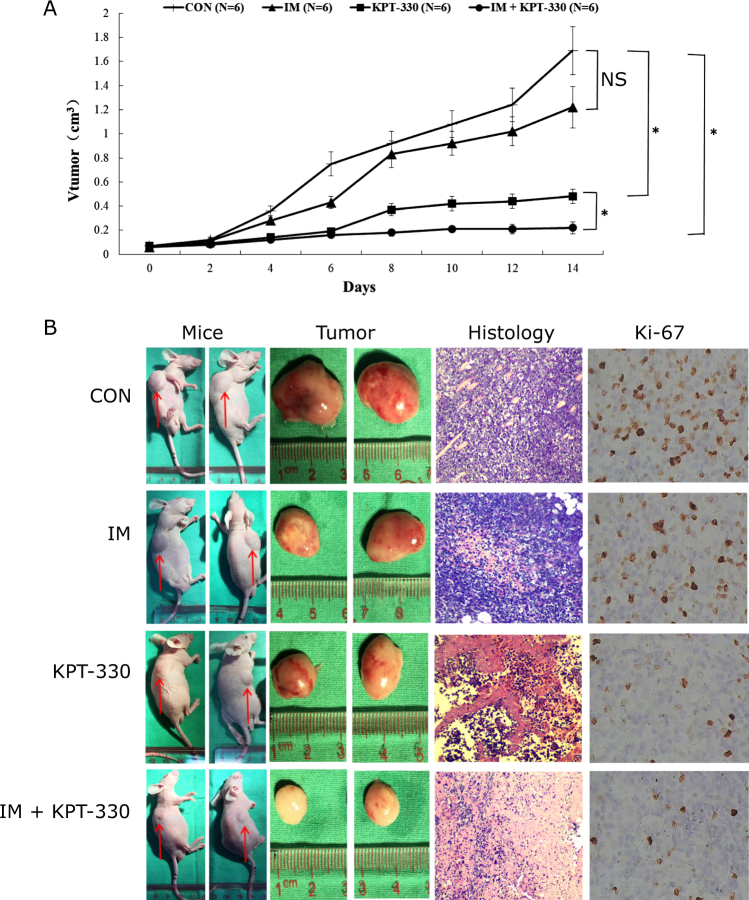
KPT-330 inhibited tumor growth and sensitized K562G to IM in mice. 2 × 10^7^ K562G cells were inoculated into the flank of right forelimb of mice. One week after inoculation, the mice were assigned into four groups (*N* = 6 each): vehicle control group, IM group, KPT-330 group, and IM + KPT-330 group. The maximum (**a**) and minimum (**b**) length of tumor were measured every 2 days. The volume of tumor was calculated by algorithm (*V*) = a × b^2^/2. After last drug-administration, mice were sacrificed and analyzed. Compared with control, the volume of tumor were smaller in KPT-330 group and IM + KPT-300 group, but not in IM group. The IM + KPT-300 combination group had much smaller tumor compared with KPT-330 alone (**a**, **b**). Data were expressed as mean ± SD. Histology analysis (×100) revealed much more necrosis and apoptosis of leukemia cells in KPT-330 group and combination group compared with IM group and control group (**b**). Ki-67 expression (×400) in KPT-330 group and combination group was significantly lower than that in IM group and control group (**b**). NS, not significant. * *P* < 0.05. One-way ANOVA, Tukey’s test

To know the anti-leukemic effect of KPT-330 in vivo, we engrafted nude mice subcutaneously with K562G cells. Seven days after inoculation, 80% (24/30) of mice developed tumor which were palpable with similar size (data not shown). Mice were treated with IM, KPT-330 alone or in combination. The volume and weight of tumor were measured. As shown in Fig. 5, compared with control, the volume of tumor were smaller in KPT-330 group and combination group (*P* < 0.05), but not in IM group (*P* > 0.05). Mean tumor weights were (0.68 ± 0.12) g and (0.39 ± 0.14) g in KPT−330 group and combination group, respectively, which were significantly lower than that in IM group (1.24 ± 0.33) g and the control group (1.54 ± 0.45) g. The inhibition of tumor for KPT-330 group and combination group was 55.84 and 74.68%, respectively, significantly higher than IM group (19.48%). Importantly, the combination group showed stronger inhibition of tumor growth compared with KPT-330 alone (*P* < 0.05). Furthermore, HE staining and immune-histochemical staining of Ki-67 of tumor tissue were performed to determine the morphology and the proliferation of leukemia cells. HE staining showed much more necrosis and apoptosis of leukemia cells in KPT-330 group and combination group compared with IM group and control group (Fig. 5b). Ki-67 expression in KPT-330 group and combination group was (32.29 ± 7.74)% and (20.77 ± 8.34)%, respectively, which were significantly lower than that in IM group (45.73 ± 9.65) and control group (52.23 ± 10.36) (Fig. 5b). Taken together, the in vivo data indicated KPT-330 inhibited tumor growth and sensitized K562G to IM in xenograft CML mouse model.

## Discussion

Although IM is a hallmark for CML treatment, emerging data have indicated that among the CML patients treated with IM, around 25% of chronic-phase patients and nearly all individuals in blast crisis acquire resistance to this drug^23^. The second generation of TKI (e.g., Dasatinib, Nilotinib) could partially overcome the drug-resistance. However, there is an increasing need for novel compounds targeting other pathways which might be used in addition to or even in place of IM or other BCR-ABL inhibitors.

In the present study, we showed the in vitro and in vivo anti-leukemic efficacy of novel inhibitor SINE KPT-330 in CML. Our data indicated that KPT-330 inhibited cell proliferation and induced cell-cycle arrest and apoptosis of CML cell lines and primary blasts, not only in the IM-sensitive, but also IM-resistant CML with BCR-ABL T315I mutation. Treatment of CML with KPT-330 caused a remarkable reduction in the amount of CRM1 and showed a significant nuclear accumulation of CRM1 cargo proteins, such as BCR-ABL and P27. In xenograft mouse model, we showed that in vivo treatment of leukemic mice with KPT-330 significantly reduced leukemic burden with little side effect.

Studies have demonstrated that CRM1 is highly expressed in a range of tumor and associated with poor prognosis and drug-resistance^24,25^. Our data showed that expression of CRM1 in CML was significantly higher than that in healthy bone marrow control. Additionally, a further higher expression of CRM1 was detected in the IM-resistant primary CML cells, compared with those was IM-sensitive. These preliminary data implied that CRM1 may present as a promising therapeutic target for CML and encouraged us to investigate the effect of CRM1 inhibitor on drug-resistant CML.

KPT-330, the orally bioavailable new generation SINE compound was used in our experiments. Although this inhibitor has exhibited clear activity in a subset of refractory/relapsed AML in ongoing clinical trials (NCT02212561, NCT02088541, and NCT02299518), very few data are available addressing its antitumor effect on CML. Here, we could clearly showed that KPT-330 was highly potent to kill cells in either IM-sensitive or IM-resistant CML cells, both in vitro and in vivo. Although it has been reported about the effectiveness of KPT-330 on a CML patient who become IM-resistant^26^, our evidence was directly obtained from the one with BCR-ABL T315I mutation, the pivotal genetic event occurring during IM-resistance. Other group showed that combination of IM and LMB had activity towards BCR-ABL positive CML cells^27^, but was still ineffective for killing BCR-ABL T315I mutant^24^. Thus, our data highlighted the activity of the novel SINE, KPT-330, in IM-resistant CML, especially those with BCR-ABL T315I mutation.

The mechanisms of drug-resistance to IM of CML include BCR-ABL dependent (gene mutations and gene amplification) and BCR-ABL independent pathways (PI3K-AKT, P27, STAT, ERK1/2 etc.)^22,28^. Located in the cytoplasm, BCR-ABL could activate PI3-AKT, promoting CML survival and associating with resistance to IM^24,29,30^. On the contrary, it has been demonstrated that translocating 30% of total BCR-ABL from the cytoplasm to the nucleus is sufficient to induce cell death^24^. We could show KPT-330 exposure led to re-location of BCR-ABL in nucleus and the subsequent inactivation of AKT, which was not observed in another publication^26^. However, in that study, total expression of BCR-ABL was significantly reduced by KPT-330, contrary to our observation as shown in Fig. 3d. This may be explained by the different cells used in the experiments (K562G CML cell line vs primary CD34 + CML and Ph + ALL), implying that the action of KPT-330 might be cell-type dependent.

Previously, we and others have demonstrated P27, the cyclin-dependent kinase inhibitor 1B, arrested the cell cycle of cancer cells and contributed to drug-resistance^3,4,31,32^. In this work, we showed that P27 was entrapped into the nucleus after exposure to KPT-330. As a result, the cell cycle of CML cells was arrested in G1 phase. This effect was observed in both IM-sensitive (K562) and IM-resistant (K562G) CML cells.

As tumor suppressor protein, STAT1 (Signal transducer and activator of transcription 1) inhibits a range of tumor growth and induces cell apoptosis^33^. The antitumor effect is correlated with the upregulation of P27 and caspase-3^34,35^. Our data showed KPT-330 enhanced the expression of p-STAT1 and caspase-3, resulting in the leukemic cell apoptosis. It remains to be elucidated how KPT-330 activated STAT1. Meanwhile, we could only detect the higher ratio of nucleus/cytoplasm, rather than the elevation of total amount of P27 after KPT-330 exposure.

Given the novel generation of ABL inhibitors with improved potency have been developed, a further direction would be the combination of KPT-330 and newer ABL inhibitors, which could abrogate the catalytic activity of all or most BCR-ABL mutants found in IM-resistant CML. In the preparation of this manuscript, the novel CRM1 inhibitor with high activity and improved tolerability, KPT-8602, has been developed and shown effectiveness in acute leukemia^36,37^. Hence, the combination of more active and selective CRM1 inhibitor with BCR-ABL inhibitor will be encouraged.

To conclude, we report here the biologic properties of CRM1 inhibitor, KPT-330, in both CML cell lines, primary CML specimens, and in a murine xenograft CML model. KPT-330 showed potent antitumor efficacy not only in common CML which were sensitive to IM, but also in refractory CML, which were resistant to IM, e.g., CML with T315I mutation. Our data indicated that KPT-330 dampened cell proliferation and induced cell-cycle arrest, and apoptosis, due to the CRM1 inhibition and its effect on cargo proteins (e.g., CRM1-BCR/ABL-AKT-P27, CRM1-STAT1-caspase 3). Given the emerging problems associated with IM monotherapy and other BCR-ABL inhibitors, our preclinical in vitro and in vivo data warrant further investigation of low-toxicity CRM1 inhibitors as a novel therapeutic alternative for refractory CML.

## Materials and methods

### Reagents

KPT-330 and IM (IM, STI571) were purchased from Selleckchem company. The antibody used for western blot, immunofluorescent staining, and immunohistochemistry were as follows: CRM1 (Santa Cruz, sc-5595), ABL pure mab (BD#554148), P27 (Abcam#32034), p-AKT (Ser473) (CST#4060), AKT (NEB#9272), p-STAT (CST#9167 S), STAT1 (CST#14995 S), caspase-3 (CST#9662), GAPDH Rabbit mAb (Genetex Chemical#HC301–02), Histone-H3 (CST#4499), Goat anti-rabbit IgG (H + L) HRP secondary antibody (Thermo Fisher #65–6120), and Ki-67 monoclonal antibody (ZSGB BIO#ZM0166).

### Cell lines and cell culture

Human CML cell lines harboring BCR-ABL chimeric oncogene, K562, and K562G which was derived from K562 and gained resistance to IM, were purchased from Institute of Hematology, Chinese Academy of Medical Sciences. All cell lines and primary CML cells were cultured in RPMI 1640 (Gibco, USA) supplemented with 10% FBS (Sijiqing, Hanzhou, China) and 100 U/ml penicillin and 80 ug/ml streptomycin.

### Primary CML samples and normal bone marrow cells

Bone marrow cells of CML patients or healthy control were obtained from Department of Hematology, Sun Yat-sen Memorial Hospital of Sun Yat-sen University, with informed consent approved by the hospital review board in accordance with the World Medical Association Declaration of Helsinki. Mononuclear cells (MNC) were acquired using density-gradient centrifugation and cultured as mentioned above.

### Cell viability assay

Cells were seeded into 96-well plates and incubated for 24, 48, and 72 h with DMSO control or KPT-330 at various concentrations as indicated. Cell viability was analyzed using the CellTiter 96 AQueous One Solution Cell Proliferation Assay (Promega #G3582). The absorbance of wells at 492 nm was measured with a microplate reader (Wellscan MK3, Labsystem Dragon USA).

### Cell-cycle analysis

Cells were incubated with DMSO control or KPT-330, harvested and fixed at a final concentration of 70% ice-cold ethanol. The cells were stored at −20 °C, washed in PBS, followed by stainning with propidium iodide/RNase A/0.01% Trizol buffer for 40 min at 37 °C. Flow cytometry analysis was performed using a FACSCalibur (BD, USA). Cell-cycle events were determined by incorporation of propidium iodide.

### Apoptosis assay

Cells were incubated and stained using the PE Annexin-V Apoptosis Detection Kit I (BD Biosciences#559763). Analysis by flow cytometry (BD, USA) was done according to the manual instruction.

### Real-time PCR

Cellular RNA was extracted using TRIzol (Invitrogen) and reverse transcribed to cDNA using ReverTra Ace qPCR RT Kit. Gene expression levels of CRM1 were detected using GoTaq® qPCR Master Mix. Normalization was performed using GAPDH expression levels. Primers were as follows: ATCTGACCCAACTTGTGTAGAGA, (H-CRM1-F),TGGTCCTACTTGCTCCAACAAT(H-CRM1-R), GAGTCAACGGATTTGGTCGT (H-GAPDH-F), and GACAAGCTTCCCGTTCTCAG(H-GAPDH-R). Comparative real-time quantitative PCR was performed in triplicate, and relative expression was calculated using the comparative Ct method.

### Western blot

Western blot was done according to standard protocol as previously described^38,39^. In brief, whole cell lysates and subcellular fractions were collected using NE-PER Nuclear and cytoplasmic protein extraction kit (Pierce). Lysates were resolved by SDS-PAGE, transferred to PVDF membrane (Millipore), followed by blocking and incubation with primary antibodies. The washed membranes were probed with HRP-conjugated secondary antibody. Membranes were developed using ECL (Pierce). Image J software was used for calculating the density of specific bands.

### Immunofluorescent staining

Cells were first counted and diluted to 1 × 10^6^/mL. Approximately 2 × 10^5^ cells were cytospun to slide. The cells were then fixed in 4% PFA for 40 min. The slides were blocked by 3% goat serum for 60 min. The cells were incubated with primary antibody overnight at 4 °C. After washing, the sample was incubated with fluorochrome conjugated secondary antibody for 1 h at room temperature. Nucleus was stained by 4,6-diamidino-2-phenylindole (DAPI). Samples were analyzed by phase contrast inverted microscope (Lecia, Germany).

### Mice

Specific pathogen-free (SPF) male BALB/c nude mice were purchased from Guangdong Medical Laboratory Animal Center (GDMLAC). All mice used in the experiments were around 4 weeks of age, with body weight of 12–14 g. All animal studies were conducted in accordance to the protocol from the Ethics Committee and regulations of the Institutional Animal Care and Use Committee at Sun Yat-Sen University.

### K562G xenograft murine model

K562G cells (2 × 10^7^) in 0.2 ml were inoculated into the flank of right forelimb of mice via subcutaneous injection. KPT-330 stock (2 mg/ml) preparation:KPT-330 50 mg + 10% Pluronic F-68 1.5 ml + PVP K-29/32 + 23.5 ml sterile water. IM stock preparation: IM diluted in sterile water for 2.33 mg/ml. One week after tumor inoculation, the mice were randomly assigned into 4 groups: vehicle control group (0.6% pluronic + PVP, oral gavage, 3 times weekly with 6 times in total; sterile water, ip, once daily with 14 times in total), IM group (0.6% pluronic + PVP, oral gavage, 3 times weekly with 6 times in total; IM 30 mg/kg, ip, once daily with 14 times in total), KPT-330 group (KPT-330 10 mg/kg, oral gavage, 3 times weekly with 6 times in total; sterile water, ip, once daily with 14 times in total), IM + KPT-330 group (IM 30 mg/kg, ip, once daily with 14 times in total; KPT-330 10 mg/kg, oral gavage, 3 times weekly with 6 times in total). The maximum (*a*) and minimum (*b*) length of tumor were measured every 2 days. The volume of tumor was calculated by algorithm (*V*) = *a* × *b*^2^/2. Twenty-four hours after last drug-administration, separate cohorts of mice were euthanized sacrificed; tumors harvested, weighed, and picture taken. Histology analysis of tumor were performed after HE staining as previously described^40^. Ki-67 expression was determined by immunohistochemistry followed manual instruction.

### Statistical analysis

Data were indicated as Mean ± SD. Differences between continuous variables (e.g., RNA expression, weight of tumor) were analyzed using *t*-tests. All *P* values were two-sided. *P* < 0.05 was considered as significant. Statistical analysis was performed by SPSS 17.0 software.
